# ADHD Prescription Medications and Their Effect on Athletic Performance: A Systematic Review and Meta-analysis

**DOI:** 10.1186/s40798-021-00374-y

**Published:** 2022-01-13

**Authors:** Jenny Berezanskaya, William Cade, Thomas M. Best, Kristopher Paultre, Carolyn Kienstra

**Affiliations:** Department of Orthopedics, University of Miami Sports Medicine Institute, 5555 Ponce De Leon Blvd, 3rd Floor, Miami, FL 33146 USA

**Keywords:** ADHD, Stimulants, Performance, Ergogenic effect, Bupropion, Methylphenidate, Adderall, Methamphetamine, Sports, Athlete

## Abstract

**Background:**

Stimulant medications used for the treatment of Attention Deficit-Hyperactivity Disorder (ADHD) are believed to provide a physical advantage in athletics, but several of these medications are not regulated by the World Anti-Doping Association. Given the prevalence of ADHD among the athlete population and concern for abuse of ADHD medications, this review and meta-analysis aimed to evaluate effects of ADHD medications on athletic performance, thereby appraising the validity of claims of performance enhancement.

**Methods:**

A search of MEDLINE, Embase, CINAHL, and Cochrane Review databases was performed for all randomized controlled trials evaluating athletic performance after ingestion of placebo or ADHD treatment medications from August 2020 through November 2020. All RCTs identified from these search criteria were included for screening, with exclusion of any animal studies. Two reviewers (JB, CK) assessed methodological quality and risk of bias using CONSORT 2010 and Cochrane Collaboration tools. Study results were compiled with corresponding p values for each finding. Effect sizes (Cohen’s D) for athletic performance and physiological changes were aggregated for each study. Studies were further screened for homogeneity that would allow for meta-analysis. Heterogeneity was calculated using I2.

**Results:**

A total of 13,033 abstracts evaluating amphetamine, methamphetamine, methylphenidate, and bupropion were screened. The final analysis included nine studies, six of which found significant improvement in athletic performance with use of stimulant medications (*p* < 0.05). Methylphenidate and amphetamine were consistently identified to have a performance effect. Secondary effects identified included significant increase in heart rate, core temperature, and elevation of various serum hormone levels (*p* < 0.05). Effect size evaluation found seven studies demonstrating small to large effects on physical performance, as well as in categories of cardiometabolic, temperature, hormone, and ratings of perceived exertion, to varying degrees. A meta-analysis was performed on two studies, demonstrating conflicting results.

**Conclusions:**

Dopaminergic/noradrenergic agonist medications appear to have a positive effect on athletic performance, as well as effects on physiological parameters. Further consideration of medications currently not regulated, i.e. bupropion, is warranted given evidence of athletic performance enhancement.

*PROSPERO trial registration number*: CRD42020211062; 10/29/2020 retrospectively registered.

**Supplementary Information:**

The online version contains supplementary material available at 10.1186/s40798-021-00374-y.

## Key Points

- All medications except methamphetamine were found to have a positive effect on measures of athletic performance.
- All medications except methylphenidate were found to have physiological effects.
- There was no conclusive effect of ADHD medications on exercise performance or power output in the meta-analysis sample, comprised of two studies.

## Background

Attention Deficit Hyperactivity Disorder (ADHD) affects 6.1 million children ages 2–17, 62% of which are treated with prescription medications [1]. The prevalence of ADHD in elite athletes is estimated at 7–8% [2], which is of particular concern given that amphetamine-based medications, such as Adderall, are banned for use with sports participation due to concern for performance enhancement. Though these medications are a crucial therapy in the treatment of many individuals with ADHD, up to 10% of high school students and 35% of college students misuse these medications for nonmedical use [3]. As such, it is critical to determine the effects of such medications in the competitive athlete population. In fact, in a study of 115 undergraduate students taking prescription ADHD medications, 31% reported misuse of the medication with the primary reason being improvement of academic performance [4]. Thus, it stands to reason that the competitive athlete is comparably, if not more, at risk of misuse of these medications if there is a potential competitive edge to be achieved.

It is postulated that ADHD pharmaceutical treatment is based on dopamine and norepinephrine agonism; however, the central nervous system (CNS) and physical impact of these medications are poorly understood. Dopamine functions to improve planning and initiation of motor responses, whereas norepinephrine improves alertness and readiness for action, among many other functions throughout the complex processes of the brain [5]. Amphetamines have been found to increase synaptic dopamine and norepinephrine levels in multiple brain regions, most notably in the striatum, while also increasing global cerebral blood flow [6]. Similarly, methylphenidate inhibits dopamine and norepinephrine transporters, thereby effectively increasing extracellular dopamine and norepinephrine, as well as activating adrenergic receptors and stimulating cortical excitability [5]. Amphetamine is the core ingredient in medications such as Adderall, Dexedrine, and Vyvanse, whereas methylphenidate is branded as Ritalin and Concerta, among others. Methamphetamine, prescribed under the name of Desoxyn, is a CNS stimulant that is metabolized to amphetamine and is approved for use in children over 6 years of age for the treatment of ADHD. It is also known to be of high risk for abuse [7]. All of these medications are banned by the World Anti-Doping Agency (WADA) [8, 9].

In recent years, bupropion, of the brand name Wellbutrin, has grown in popularity for the off-label treatment of ADHD [8]. Used as a non-stimulant alternative, bupropion has been cited in the 2021 Monitoring Program by WADA, but is not considered a prohibited substance [9]. This medication exerts its effects by inhibiting dopamine and norepinephrine reuptake [10] and thus is grouped with the aforementioned stimulant medications through its ultimate effects of increasing dopamine and norepinephrine.

Additional medications used for the treatment of ADHD include atomoxetine, guanfacine and clonidine, all of which are not prohibited by WADA [9]. Atomoxetine was found to enhance norepinephrine and dopaminergic activity threefold in the prefrontal cortex [11]. Similarly, both guanfacine and clonidine increase norepinephrine activity specifically in the prefrontal cortex, which is related to improving working memory and attention [12]. It is conjectured that these medications do not impact motor function, since their effect is limited to the prefrontal cortex, whereas medications altering striatal neurotransmitter concentrations are associated with motor behavior regulation [11]. Even so, in rats, guanfacine was found to increase swimming speed [13], thereby confirming that the potential ergogenic effect of these medications is poorly understood. Research in humans is lacking for all three of these medications.

Though the potential for stimulant medications to cause harm is the predominant reason for their avoidance in the athletic population, most sporting organizations allow use with a documented diagnosis and compliance with appropriate reporting procedures [8]. The belief that these medications provide a physical advantage has been purported but yet to be conclusively validated in humans. However, in animal models, rats treated with amphetamine were able to run significantly longer than non-treated controls [14]. More specifically, rats treated with amphetamines demonstrated increased time to exhaustion, increased time to reach VO_2_max with concomitant increase in VO_2_max values, and higher core temperatures at exhaustion, suggesting that amphetamines decrease the sensation of fatigue [15]. These findings appear to support the assertion that ADHD medications have the potential to enhance human physical capabilities.

To date, there has been no critical appraisal of the literature assessing athletic performance effects of all available prescription medications that increase CNS dopamine/norepinephrine concentrations, as discussed above. Therefore, we performed a systematic review and meta-analysis to evaluate the available literature on all medications used to treat ADHD and their measurable effects on athletic performance. Secondary endpoints included any medication-specific or dose effects, heart rate differences, core temperature variability, and changes in hormonal concentrations. While the intended purpose of these medications is to improve concentration and focus, the aim of this review was to objectively assess any potential for ergogenic effect and any corresponding physiological changes that may result in an adverse effect.

## Methods

### Search Strategy and Inclusion Criteria

The protocol was registered at PROSPERO (CRD42020211062). A search of MEDLINE, Embase, CINAHL, and Cochrane Review databases was performed for all randomized controlled trials (RCTs) evaluating athletic performance after ingestion of placebo or ADHD treatment medications from August 2020 through November 2020. Medication keywords “amphetamine”, “methamphetamine”, “methylphenidate”, “ritalin”, “bupropion”, “adderall”, “vyvanse”, “dopamine norepinephrine”, “atomoxetine”, “guanfacine”, “clonidine”, and “stimulant” were utilized in combination with “athlete performance” or “sports” or “athlete”. All RCTs identified from these search criteria were included for screening, with exclusion of any animal studies. Duplicates were removed, and remaining full text studies were reviewed.

### Methodological Quality and Risk of Bias Assessment

Two reviewers (JB, CK) assessed methodological quality and risk of bias using CONSORT (Consolidated Standards of Reporting Trials) 2010 and Cochrane Collaboration tools, respectively. CONSORT scores out of a maximum of 25 points were averaged, and discrepancies of  > 2 points were resolved by a third reviewer (KP). For assessment of risk of bias, two reviewers (JB, CK) independently evaluated each study.

### Data Extraction and Analysis

Data from each study were extracted to Microsoft Excel, tracking first author and year of publication, study demographics, and results. Study demographics included study design, participant sample size, age and sex distribution, level of athleticism, medication used, and performance measure. Study results were compiled, including performance effects, physiologic changes including heart rate, core temperature, and VO_2_max effects, as well as hormone differences, with corresponding p values for each finding. Effect sizes (Cohen’s D) for athletic performance and physiological changes identified were collected and/or calculated for each study. Following convention, effect sizes were categorized as null (< 0.2), small (0.2–0.4), moderate (0.5–0.7), or large (0.8 or greater).

Studies were further screened for homogeneity that would allow for meta-analysis. Heterogeneity was calculated using *I*^2^; fixed effect models were used when heterogeneity was low to moderate (*I*^2^ < 50%), and random effects models were used when heterogeneity was high (*I*^2^ ≥ 50%). For the outcomes of interest, two separate absolute statistics (i.e. standardized mean difference) were presented and 95% confidence intervals were determined. A *p* value of 0.05 was considered significant.

## Results

### Study Selection

**Fig. 1:**
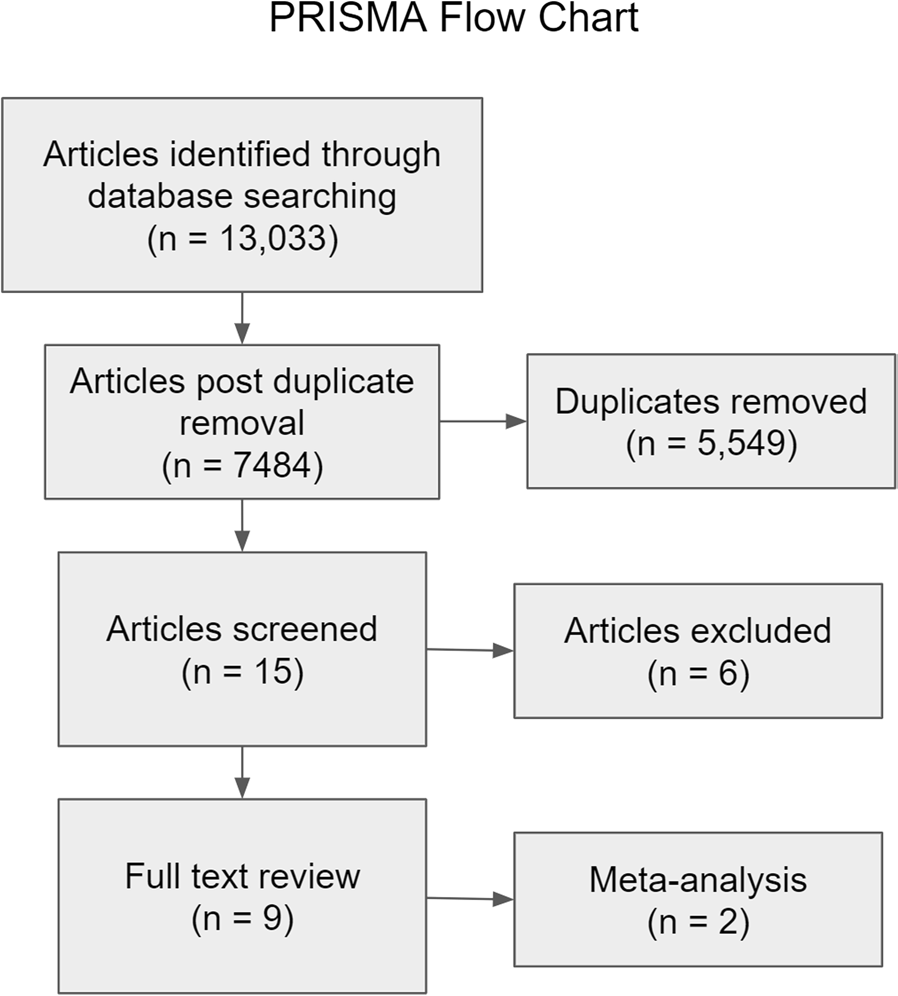
PRISMA flow chart

The search yielded 13,033 potential reports. Upon removal of 5549 duplicates, 7484 studies remained for screening based on title and subsequently by abstract. A total of fifteen articles were then screened in full text and assessed for eligibility. The average CONSORT score among all reports found was utilized as a baseline to determine study acceptability. The average score of all reports was 12.65. Six studies were excluded due to a CONSORT score of < 12.5, with the conclusion that nine studies were appropriate for analysis. PRISMA flow chart is presented in Fig. 1.

### Assessment of Methodologic Quality and Risk of Bias

The nine studies that were included in the final analysis had a mean quality score of 13. One study demonstrated high selection bias, while eight did not provide adequate information to determine allocation concealment, random sequence generation, and detection bias. All nine studies showed low attrition and reporting bias. Table 1 provides detailed bias assessment for each study.

**Table 1:** Bias assessment

Study	Random sequence generation	Allocation concealment	Blinding of participants and personnel	Blinding of outcome assessment	Incomplete outcome data	Selective reporting	Other sources of bias
Dufka et al. [16]	(?)	(?)	(−)	(?)	(−)	(−)	(−)
King et al. [19]	(?)	(?)	(−)	(?)	(−)	(−)	(−)
Roelands et al. [20]	(?)	(?)	(−)	(?)	(−)	(−)	(−)
Cordery et al. [21]	(?)	(?)	(−)	(?)	(−)	(−)	(−)
Mahon et al. [24]	(+)	(+)	(+)	(+)	(−)	(?)	(?)
Piacentini et al. [22]	(?)	(?)	(−)	(?)	(−)	(−)	(−)
Roelands et al. [23]	(?)	(?)	(−)	(?)	(−)	(−)	(−)
Chandler and Blair [17]	(−)	(?)	(−)	(?)	(−)	(−)	(−)
Altszuler et al. [18]	(?)	(?)	(−)	(−)	(−)	(−)	(−)

Summary of bias assessment findings, with (+) indicating high risk, (−) indicating low risk, and (?) indicating unclear risk

### Study Demographics and Characteristics

Across the nine studies, there were a total of 157 participants, 111 of which were male and 34 were female; one study did not provide sex data. Evaluation of medication types was as follows: one methamphetamine study, three methylphenidate studies, three bupropion studies, one amphetamine study, and one mixed methylphenidate or amphetamine study. There were no studies found evaluating performance effects of atomoxetine, guanfacine, or clonidine that met inclusion criteria. Participant demographics included children with ADHD diagnoses, college students with no indication of level of fitness, average citizens who participated in regular endurance exercise, and trained cyclists. Study design, demographics, and performance measures are reported in Table 2.

**Table 2:** Study characteristics

Study	Study design	Blinding	Patients (M:F)	Level of fitness/study population	Medication dose	Dose timing	Groups (*N* =)	Age	Dropout rate	Performance measures
Dufka et al. [16]	RCT	Double blind, randomized	12 (sex breakdown unknown)	High school students	L-methamphetamine 16, 48 mcg	Immediately prior to test	L-methamphetamine 4 or 12 inhalations (*N* = 12)	14–17 (no mean provided)	0	Miles travelled in 20 min on stationary bike
							Placebo (*N* = 12)			
Altszuler et al. [18]	RCT	Double blind, randomized	54:19	Kids with ADHD diagnoses in a summer training program	Methylphenidate 21 mg	Daily for 3 weeks, then for final 3 weeks also attended sports training program for badminton	Methylphenidate (Concerta or Focalin) (*N* = 37)	7.99 ± 1.7	0	Playing badminton (sports skills in underhands, backhands, overheads, serves; sport knowledge; staff ratings from summer treatment program counselors; behavioral assessment in rule violations, game awareness, name-calling peers, verbal abuse to staff, and complaining)
							Placebo (*N* = 36)			
King et al. [19]	RCT, crossover	Double blind	9:6	Unknown; right—handedness required	Methylphenidate IR 20 mg	Prior to test (time not specified)	Methylphenidate 20 mg (*N* = 15)	28.4 ± 5.2	0	Handgrip task
							placebo (*N* = 15)			
Roelands et al. [20]	RCT, crossover	Double blind, randomized	8:0	Well-trained cyclists	Methylphenidate 20 mg	1 h prior to test	Methylphenidate 20 mg (*N* = 8)	26 ± 5	0	Cycling time to complete a timed trial at 18C and 30C
							Placebo (*N* = 8)			Core temperature, blood (cortisol, growth hormone, ACTH, beta-endorphin, hematocrit)
Cordery et al. [21]	RCT, crossover	Double blind, randomized	0:9	Active, participate in regular endurance exercise training	Bupropion 150 mg × 4	Before bed the night before and upon wakening morning of test	Bupropion 150 mg (*N* = 9)	21 ± 2	0	Cycle at 60% VO2peak for 60 min, followed by 30 min performance test, at 30C
							Placebo (*N* = 9)			Core temperature, O_2_ consumption, CO_2_ production, respiratory exchange ratio
Piacentini et al. [22]	RCT, crossover	Double blind, randomized	8:0	Well trained cyclists	Bupropion 600 mg	Taken night before and 6 h before test	Bupropion (*N* = 8)	24 ± 2	0	Maximal power output, endurance performance tests (timed trials)
							Placebo (*N* = 8)			VO_2_ max, HR, ACTH, prolactin, cortisol, growth hormone, beta-endorphins, catecholamines
Roelands et al. [23]	RCT, crossover	Double blind, randomized	8:0	Trained cyclists	Bupropion 150/300 mg	Bupropion 150 × 3 days daily, then 300 mg × 7 days (150 mg in am and in pm)	Bupropion 150/300 mg (*N* = 8)	27 ± 5	0	Fixed cycle intensity exercise for 60 min followed by timed trial, at 30C
							Placebo (*N* = 8)			Core temperature, HR, hormones (growth hormone, cortisol, ACTH, prolactin), rating of perceived exertion
Chandler and Blair [17]	RCT	Double blind, randomized	6:0	College students, former high school athletes	Amphetamine sulfate 15 mg per 70 kg of bodyweight (mean dose 16.59 mg)	Ingestion 2 h and 5 h before testing	Dexedrine (*N* = 6)	21.5 ± 2.5	0	Strength (elbow flexion and knee extension strength), muscular power (bicycle ergometer, in total time to execute 5 revolutions), running speed (30 yard sprint time), acceleration, time to exhaustion
							Placebo (*N* = 6)			HR, respiratory exchange ratio, lactic acid, aerobic power, aerobic capacity (VO_2_max)
Mahon et al. [24]	Controlled trial	Unblinded, unrandomized	18:0	Average, prior diagnosis of ADHD required	Varied dose and medication type	12–24 h prior to test and 1–2 h prior to test	Methylphenidate or amphetamine (*N* = 14)	10.9 ± 1.1	4	Cycle ergometer at 25, 50, and 75 W for 3 min each
							Placebo (*N* = 14)			Heart rate, peak VO_2_, Ve/VO_2_, respiratory exchange ratio, rating of perceived exhaustion

### Medication-Specific Performance Effects

Study-specific findings are recorded in Table 3. In sum, six reports found significant improvement in athletic performance with use of stimulant medications, with detailed review of respective *p *values provided below.

**Table 3:** Performance changes summary

Study	Medication	Performance measure	Performance effect	
Dufka et al. [16]	Methamphetamine	Cycling	No effect on distance travelled (*p* = 0.81)	(−)
Altszuler et al. [18]	Methylphenidate	Badminton	Medication led to improved performance in knowledge in recreation condition. Only sportsmanship and effort improved on medication by counselor rating (*p* < .01). Sports training and medication both significantly improved rule violations (*p* < .05, *p* < .001)	(+)
King et al. [19]	Methylphenidate	Handgrip	Mean force over all trials significantly higher in methylphenidate group (*p* = 0.032)	(+)
Roelands et al. [20]	Methylphenidate	Cycling	Methylphenidate increased exercise performance in warm conditions (*p* = 0.049). Power output was greater in methylphenidate group (*p* = 0.028)	(+)
Cordery et al. [21]	Bupropion	Cycling	Total work significantly higher in bupropion trial (7.5 ± 9.6% increase; *p* = 0.042)	(+)
Piacentini et al. [22]	Bupropion	Cycling	No difference in exercise performance (time to compete target amount of work)	(−)
Roelands et al. [23]	Bupropion	Cycling	No significant differences in timed trial or max power output in bupropion trial versus placebo	(−)
Chandler and Blair [17]	Amphetamine	Multiple (cycling, running, strength)	Acceleration (*p* < 0.05), knee extension strength (*p* < 0.01), anaerobic capacity (*p* < 0.05), time to exhaustion (*p* < 0.01), all significantly increased	(+)
Mahon et al. [24]	Mixed	Cycling	Work rate (exercise intensity) at peak exercise significantly higher with medication (*p* < 0.05)	(+)

Summary of findings, with (+) indicating a significant difference identified and, (−) indicating no significant difference found

#### Methamphetamine and Amphetamine

The use of an inhaled version of L-methamphetamine did not demonstrate a significant change in distance travelled during cycling trials with use of 16mcg or 48mcg dose (*p* = 0.81) [16]. Conversely, amphetamine, at a dose of 15 mg per 70 kg bodyweight, was evaluated via cycling and running trials, with findings of increase in acceleration (*p* < 0.05), knee extension strength (*p* < 0.01), and time to exhaustion (*p* < 0.01) [17].

#### Methylphenidate

Performance effects of methylphenidate were tested via badminton skill acquisition [18], handgrip strength [19], and cycling timed trials [20]. Study participants provided with 21 mg of methylphenidate were found to have improvement in sportsmanship and effort (*p* < 0.01) but not in acquisition of sport-specific skills [18]. King et al. [19] and Roelands et al. [20] both utilized 20 mg methylphenidate in their studies. The methylphenidate groups demonstrated significantly greater mean force in handgrip strength (*p* = 0.032) [19]. In cycling trials, methylphenidate groups finished 16% faster (*p* = 0.049) and had significantly greater power output (*p* = 0.028) [20].

#### Bupropion

One of three studies testing bupropion’s effect on performance found significant improvement in work done in cycling trials (*p* = 0.042), citing a 7.5 ± 9.6% increase with use of 600 mg of bupropion [21]. Even so, two studies evaluating three different doses of bupropion (150 mg, 300 mg, and 600 mg) found no significant difference in exercise performance by way of time to complete a target amount of work or in maximum power output [22, 23].

#### Mixed Medications

One study evaluated physical performance changes among children with established ADHD diagnoses and already on treatment with methylphenidate or amphetamine. An overall increase in work rate was found in participants taking these medications (*p* < 0.05) [24].

#### Effect Size

To better ascertain the clinical significance of each study’s findings, effect sizes were analyzed for each performance measure (Table 4). All medications tested demonstrated an effect on physical performance. No effect was found for two of three bupropion studies, with the third demonstrating small to moderate effect on physical performance. Performance measures associated with the largest positive effect included: exercise performance in a timed trial, power output, and knee extension strength. Methylphenidate had a small to large effect in all performance parameters, whereas amphetamine had predominantly small effect in parameters measured. There was no performance measure that was found to consistently demonstrate small to large effect size among all medications evaluated.

**Table 4:** Physical performance effect sizes

Study	Medication	Performance measure	Effect size	
Dufka et al. [16]	Methamphetamine	Distance travelled	0.07–0.11	Null
Altszuler et al. [18]	Methylphenidate	Sport skills/abilities	0.42	Small
		Effort	0.74	Moderate
King et al. [19]	Methylphenidate	Increase in mean force	0.162	Small
Roelands et al. [20]	Methylphenidate	Increased exercise performance (timed trial)	1.063	Large
		Increased power output	0.833	Large
Cordery et al. [21]	Bupropion	Total work increase	0.0468	Null
Piacentini et al. [22]	Bupropion	Time to compete target amount of work	0	Null
Roelands et al. [23]	Bupropion	Increased exercise performance (timed trial)	0.27	Small
		Max power output increase	0.69	Moderate
Chandler and Blair [17]	Amphetamine	Elbow flexion strength	0.45	Small
		Muscular leg power	0.26	Small
		Acceleration	0.43	Small
		Knee extension strength increase	1.27	Large
		Speed	0	Null
		Time to exhaustion increase	0.43	Small
Mahon et al. [24]	Mixed	Work rate (exercise intensity) increase	0.63	Moderate

### Analysis of Secondary Effects

Through the course of the review of the literature, several secondary effects of medication use were noted to be consistently evaluated, namely in the categories of cardiometabolic effects (i.e. changes in heart rate, blood pressure, oxygen consumption, plasma glucose levels, or plasma lactate levels), core temperature, hormone changes, and ratings of perceived exertion or thermal stress. Table 5 summarizes secondary effects of medication treatment per study. Where the data were available, effect sizes were aggregated and/or calculated,  which are subsequently summarized in Table 5.

**Table 5:** Secondary effects summary

Study	Medication	Temperature effect		Cardiometabolic effects		Hormone effects		Ratings of perceived exertion (RPE) and thermal stress		Effect size		
Dufka et al. [16]	Methamphetamine	–	n/a	No significant effect on BP, subjective perception of performance, energy, endurance or ability to breathe	(−)	–	n/a	–	n/a	Systolic BP	0.07–0.34	Null to Small
										Diastolic BP	0.13–0.77	Null to Large
										Heart Rate	0.01–0.57	Null to Moderate
Altszuler et al., [18]	Methylphenidate	–	n/a	–	n/a	–	n/a	–	n/a	n/a		
King et al. [19]	Methylphenidate	–	n/a	–	n/a	–	n/a	–	n/a	n/a		
Roelands et al. [20]	Methylphenidate	Core temperature increased significantly following methylphenidate at 30C (*p* = 0.013), but no temperature effect found at 18C (*p* = 0.360). Core temp rose higher in methylphenidate 30, significant after 25 (*p* = 0.006) and 30 min (*p* = 0.006) and at end of timed trial (*p* = 0.014). During recovery, core temp was higher at all time points in methylphenidate (*p* 0.009, 0.011, 0.018)	(+)	Significant increase in heart rate at every time point during timed trial post methylphenidate at 30C (*p* = 0.046) (no difference apparent at 18C)	(+)	No difference in blood hormone (cortisol, ACTH, GH, beta-endorphin) concentrations (all hormones measured increased during exercise in all trials, but no differences with drug treatment)	(−)	No difference in rating of perceived exertion or thermal stress	(−)	n/a		
Cordery et al. [21]	Bupropion	No significant difference in core temperature increase (*p* = 0.579). Core temperature higher in bupropion trial only at last 5 min of exercise test when participants gave all-out effort (*p* = 0.021)	(+)	No significant difference in heart rate, but significantly higher in bupropion group in last 5 min with max effort (*p* = 0.043). No differences in sweat loss. No significant difference in VO_2_	(+)	Slight but statistically significant increase in plasma testosterone before the bupropion trial compared to placebo (*p* = 0.043). Prolactin was elevated at end of exercise in bupropion group (*p* = 0.039)	(+)	No significant difference in ratings of perceived exertion or expired gases. No differences in rating of thermal sensation between groups	(−)	Heart rate	0.537	Moderate
										Core Temperature	0.588	Moderate
										Cortisol	0.03	Null
										Prolactin	0.35	Small
										FSH	0.57	Moderate
										LH	0.11	Null
Piacentini et al. [22]	Bupropion	–	n/a	No significant difference in lactate concentration and HR	(−)	Cortisol significantly higher in bupropion group at rest, at 60 min, at end of exercise (*p* < 0.05). ACTH significantly higher in bupropion group only at end of exercise (*p* < 0.05)	(+)	No difference in RPE among trials	(−)	Heart rate	0.36	Small
										Lactate Concentrations	0.2	Small
										RPE	2.43	Large
Roelands et al. [23]	Bupropion	Total core temperature significantly higher in bupropion trial compared to placebo (*p* = .030)	(+)	No significant difference in heart rate among groups	(−)	Growth hormone significantly higher in bupropion group (*p* = .008). No difference in prolactin cortisol, ACTH	(+)	No difference in ratings of perceived exertion among groups	(−)	Heart Rate	0.56	Moderate
										Core Temperature	1	Large
										Weighted skin temperature	1.18	Large
										Thermal stress scores	1.81	Large
Chandler and Blair [17]	Amphetamine	–	n/a	Maximum heart rates significantly increased (*p* < 0.001)	(+)	–	n/a	–	n/a	VO_2_Max	0.02	Null
										Anaerobic Capacity	0.58	Moderate
										Pre-exercise heart rate	0.72	Moderate
										Maximum heart rate	0.55	Moderate
										Respiratory exchange ratio	0	Null
Mahon et al. [24]	Mixed	–	n/a	No effect on Ve/VO_2_, RER. Heart rate significantly higher with medication compared to placebo (*p* < 0.05). Dose of medication and heart rate demonstrated no statistically significant correlation. VO_2_ significantly higher with medication at peak exercise (*p* < 0.05)	( +)	–	n/a	No effect on RPE	(−)	VO2 (oxygen uptake)	0.09–0.58	Null to Moderate
										VE/VO_2_ (ventilatory equivalent for oxygen)	0.38–0.48	Small to Moderate
										Respiratory exchange ratio	0–0.26	Null to Small
										Heart rate	0.78	Large
										RPE	0.03–0.34	Null to Small

Summary of findings, with (+) indicating a significant difference identified and, (−) indicating no significant difference found. N/A indicates no data to report

#### Cardiometabolic

Seven studies evaluated multiple metabolic factors, including blood pressure, heart rate, VO_2_max, respiratory exchange ratio, ventilatory equivalent for oxygen, and lactate levels. Heart rate was elevated in the medication groups in four studies, representing methylphenidate (*p* = 0.046) [20], bupropion (*p* = 0.043) [21], amphetamine (*p* < 0.001) [17], and mixed medications (*p* < 0.05) [24]. Peak VO_2_ was found to be significantly higher in only one of two studies, in which mixed medications were used (*p* < 0.05) [24]. No significant differences were identified in all other parameters examined. Of the seven studies, six demonstrated small to moderate effect on all parameters [16, 17, 21–24], while for one study [20], the effect sizes could not be calculated with the data provided.

#### Core Temperature

Core temperature was tracked in three studies, all of which found significant increase in core temperature after medication treatment. Specifically, core temperature increased significantly with methylphenidate treatment (*p*= 0.013) at all time points measured during the study [20]. Similarly, core temperature was higher with bupropion treatment in Cordery et al. [21] (*p* = 0.021) and Roelands et al. [23] (*p* = 0.030) studies. Two studies provided data to allow for evaluation of effect size and demonstrated moderate to large effect on core temperature with use of bupropion [21, 23].

#### Hormones

Four studies examined change in hormone concentration with medication treatment. Three studies found significant increase in hormone concentration after medication treatment, all of which were after treatment with bupropion. Specifically, prolactin (*p* = 0.043) [21], cortisol (*p* < 0.05) and ACTH (*p* < 0.05), and growth hormone (*p* = 0.008) [23] were all increased in the bupropion treatment groups. Only one study provided sufficient data for evaluation of effect size and demonstrated small effect on prolactin increase and moderate effect on FSH increase but no effect on cortisol or LH increase with bupropion use specifically [21].

#### Ratings of Perceived Exertion and Thermal Stress

Five studies examined ratings of perceived exertion and/or thermal stress, none of which found any significant differences between placebo and medication treatment groups. Effect sizes for three of these studies were able to be calculated, with finding of large effect with bupropion use in two studies [22, 23]. There was varied effect size in a second study which utilized multiple different stimulant medications [24].

### Meta-analysis

A meta-analysis was completed for both Roelands et al. [20, 23] studies, as these studies utilized identical experimental designs. Analysis of variance for exercise performance and power output with two-tailed *F*-test was performed. The null hypothesis was that methylphenidate and bupropion do not impact exercise performance and power output, independently. The calculated *F* = 1.41 for exercise performance and calculated *F* = 1.358 for power output were both less than the *F* statistic of 4.99, indicating that the null hypothesis could not be rejected.

**Fig. 2:**
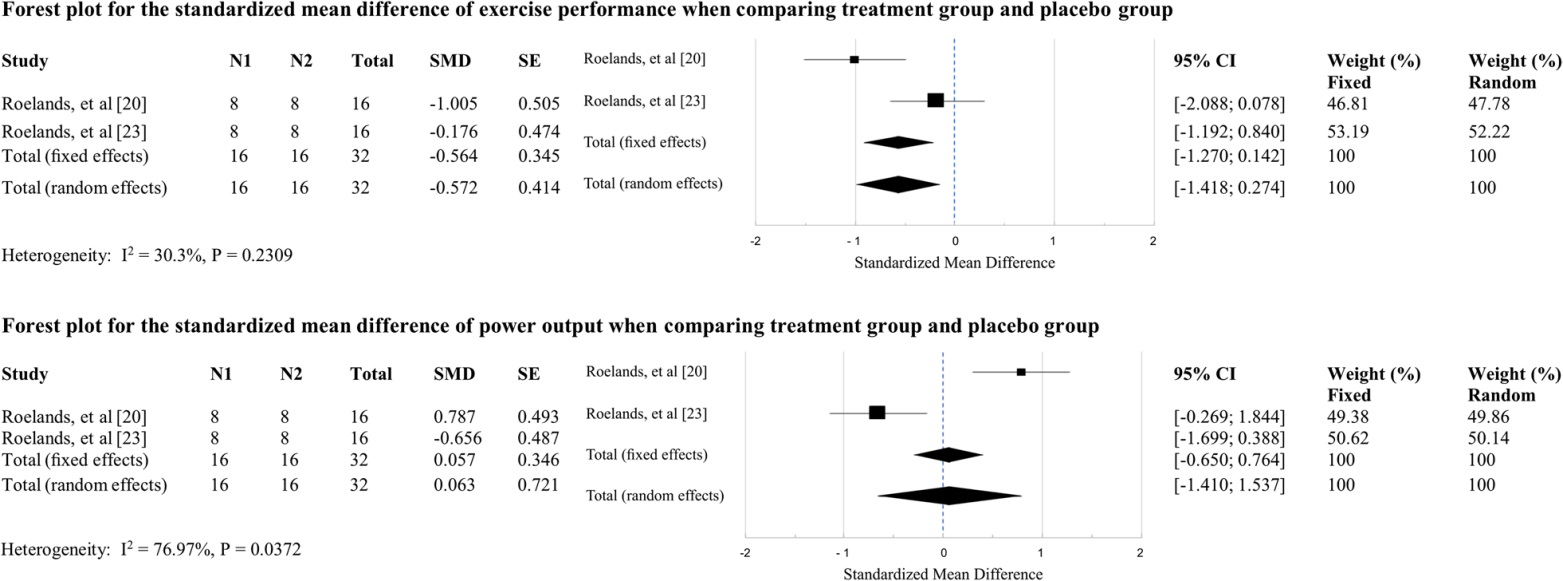
Meta-analysis of exercise performance and power output

The data were pooled and standardized mean differences were calculated for exercise performance and power output, among the treatment and placebo groups. Forest plots for exercise performance and power output were generated for the two standardized mean differences (Fig. 2). Heterogeneity was minimal for exercise performance (*I*^2^ = 30.3, *p* = 0.2309) but extensive for power output (*I*^2^ = 76.9, *p* = 0.0372), and not explained by any single study.

## Discussion

The primary goal of this systematic review was to evaluate evidence demonstrating athletic performance enhancement due to use of medications that treat ADHD. In an effort to be as inclusive as possible, all medications currently available for treatment of ADHD were evaluated; however, due to paucity of data available, this systematic review excluded atomoxetine, clonidine, and guanfacine. Even so, based on our analysis, there is support for the presumption that stimulant medications impact athletic performance. This is particularly evident in evaluation of effect sizes, such that all but one medication demonstrated an effect on athletic performance. Specifically, methylphenidate, bupropion, and amphetamine all demonstrated an effect on measures of physical performance in terms of power output and exercise performance in timed trials. Methamphetamine did not demonstrate performance effects, although there was only one study available for evaluation of methamphetamine and performance impact. No specific measure was found to consistently demonstrate null effect. Even so, the variety of measures demonstrating small to large effects, regardless of the medication, provides added reason for their monitoring and regulation in the athletic population.

These findings suggest that the effects of ADHD medications are not limited strictly to attentional improvement. By acting on the CNS, there appears to be a peripheral benefit, even though there is no evidence of stimulant medications acting on the peripheral nervous system. Stimulant effects on concentration alone cannot explain increases in strength and level of exertion. It is possible that perception of fatigue may play a role, but more research is necessary in order to validate this claim, as well as to determine the mechanism by which stimulant medications provide physical benefit.

In an effort to determine if ADHD medication use was associated with any adverse effects, particularly given that a number of these medications are banned due to fear that they may do so, an analysis of physiological effects was completed. There were no adverse events reported in any study included for review. Typical side effects of stimulant medications are mild and temporary, including but not limited to: decreased appetite, trouble sleeping, weight loss, increased heart rate, increased blood pressure, abdominal pain, and headache [25]. Notably, several studies reported increases in heart rate, core temperature, and hormone concentrations with these medications. Heart rate was consistently found to be increased with medication treatment compared to placebo groups, but the significance of this in determining the safety of these medications for use during sports participation is yet to be elucidated. In the setting of stimulant medication abuse and excessive use, these physiological effects have the potential to result in significant adverse effects.

The two studies utilized for the meta-analysis had identical protocols but used two different medications: methylphenidate [20] and bupropion [23]. Conversely, their effect sizes (by way of SMD and forest plots) for both exercise performance and power output are contradictory; methylphenidate demonstrated significant positive effect on power output but significant negative effect on exercise performance, and the converse was true of bupropion. Additionally, the power output condition demonstrated high heterogeneity, which calls into question whether the effects found are truly due to the medication. This, together with the results of the analysis of variance, ultimately leads us to infer that the results of the meta-analysis are inconclusive.

### Limitations

This review is not without its limitations. First, the premise of grouping these medications relies upon the available data on the mechanism of action of each medication, specifically on the assertion that they affect norepinephrine and dopamine levels within the CNS. It is important to note that the physiological effect of all medications used to treat ADHD is poorly understood and thus may be subject to change. In general, there are few studies for any individual medication, thus making it difficult to determine any single medication’s effect. For instance, bupropion was found to have a performance effect in one of three studies, thus calling into question the significance of this finding. Yet, in the interest of monitoring the safe and fair use of these medications in the competitive athlete population, this result should not be disregarded.

Furthermore, this review excluded evaluation of three medications (atomoxetine, clonidine, guanfacine) used to treat ADHD and thus cannot determine their role in athletic performance. This is particularly notable given these medications have a primary effect in the prefrontal cortex, whereas those included in this review affect the striatum preferentially. How these differences in CNS activation correlate with physical performance is thus unclear.

An additional confounding factor is that two studies [18, 24] included a population of children with ADHD and already on stimulant medications, whereas all other studies included participants who were naive to stimulant medications. Though these studies demonstrated up to moderate effect on physical performance, it is unclear how the chronicity of stimulant medication use impacts athletic performance overall. The dosing of stimulant medications, acutely and chronically, is of particular interest in the setting of their abuse and thus warrants further research.

In further evaluation of each report, the sample sizes amongst each individual study were all small, thereby limiting generalization to the greater population. This was particularly the case for the meta-analysis given its inclusion of only two studies. Vast variability among study designs led to difficulty in finding sufficient similarities between studies. Furthermore, the general paucity of research available on the performance effects of stimulant medications further limited evaluation of these medications. Additionally, the study findings may not be applicable to older populations given the ages of the study participants. The majority of participants were also male, thereby limiting our ability to ascertain any sex-specific effects. Likewise, dose-specific alterations to athletic performance are unclear based on the results of this review, as is timing of medication dosing. It is notable that some of these medications require an extended period of time of consistent use prior to resulting in any benefit. With the exception of one report [24], all included studies utilized the medications prior to testing, and thus any potential long-term ergogenic benefit was not assessed. The great variability of experimental design, population, and performance measures, and physiological testing ultimately creates difficulty in thoroughly assessing any one medication’s effect on athletic performance and any negative effects, but underpins the importance of the CNS in regulating physical activity.

Elucidation of the effect of these medications throughout the body is needed, though practically difficult due to the need for extensive resources in order to conduct proper testing. One must also consider the complexities of patient compliance, testing in various populations, and ensuring safe practices, in order to answer the many questions that there still are to answer on this subject. Further research is warranted and should focus on establishing dose specific effects and adverse events, as well as elucidating medication mechanisms of action and differences in CNS stimulation as correlated with physical performance changes.

## Conclusion

This systematic review suggests that medications increasing dopamine and norepinephrine concentrations within the CNS may provide a measurable performance benefit. Additionally, physiological effects are apparent, particularly with regard to cardiometabolic changes. Clinically, these results support the need for regulation of amphetamine-based medications and consideration for oversight of bupropion use, given this medication is not currently prohibited in athletic competition. Based on available research, recommendations for monitoring of atomoxetine, guanfacine, and clonidine cannot be made.

## Supplementary Information

**Additional file 1**. Boolean terms utilized for database searches.

## Data Availability

All data relevant to the systematic review and meta-analysis are included in the article or available as Additional file 1.
