# Supplementary material for: ADHD Prescription Medications and Their Effect on Athletic Performance: A Systematic Review and Meta-analysis
Source: Sports Med Open. 2022 Jan 13;8:5. doi: 10.1186/s40798-021-00374-y (PMC8755863; doi:10.1186/s40798-021-00374-y)
Supplement: Supplementary file 1 — Additional file 1. Boolean terms utilized for database searches. [file 40798_2021_374_MOESM1_ESM.docx]

Boolean terms utilized:

MEDLINE:

(((((((((((((athlete[MeSH Terms]) OR (athletic performance[MeSH Terms])) OR (sports[MeSH Terms])) AND (amphetamine[MeSH Terms])) OR (methamphetamine[MeSH Terms])) OR (stimulant[MeSH Terms])) OR (vyvanse[MeSH Terms])) OR (ritalin[MeSH Terms])) OR (bupropion[MeSH Terms])) OR (adderall[MeSH Terms])) OR (dopamine norepinephrine[MeSH Terms])) OR (atomoxetine[MeSH Terms])) OR (guanfacine[MeSH Terms])) OR (clonidine[MeSH Terms])​​

Embase:

((((amphetamine OR methamphetamine OR methylphenidate OR stimulant OR dopamine) AND norepinephrine OR ritalin OR bupropion OR adderall OR vyvanse) AND sports OR sports) AND performance OR athletic) AND performance AND randomized AND controlled AND human NOT animal AND 'randomized controlled trial'/de AND 'article'/it​

Cochrane:

"amphetamine" or "methamphetamine" or "methylphenidate" or "stimulant" or "dopamine norepinephrine" or "ritalin" or "bupropion" or "adderall" or "vyvanse" or "atomoxetine" or "guanfacine" or "clonidine" in Title Abstract Keyword AND "athlete" or "athletic performance" or "sports" in Title Abstract Keyword NOT caffeine in Title Abstract Keyword

CINAHL:

amphetamine OR methamphetamine OR methylphenidate OR vyvanse OR adderall OR ritalin OR dopamine norepinephrine OR guanfacine OR atomoxetine OR clonidine OR stimulant AND sports AND athlete performance
